# Comparing supervised and semi-supervised Machine Learning Models on Diagnosing Breast Cancer

**DOI:** 10.1016/j.amsu.2020.12.043

**Published:** 2021-01-08

**Authors:** Nosayba Al-Azzam, Ibrahem Shatnawi

**Affiliations:** a Department of Physiology and Biochemistry, Faculty of Medicine, Jordan University of Science and Technology, Irbid, 22110, Jordan; b Independent Researcher in Data Analytics, Jordan

**Keywords:** Diagnosis, Machine learning algorithms, Semi-supervised, Supervised, Breast cancer, KNN, K- nearest neighbor, MRI, Magnetic resonance imaging, Xgboost, eXtreme Gradient Boosting, SVM, Support vector machine, SSL, Semi-Supervisd Learning, SL, Supervised Learning, RBF, Radial Basis Function, ID3, Information Gain, WDBC, Wisconsin Diagnostic Breast Cancer, FNA, fine needle aspirate, EDA, Exploratory Data Analysis, Cov, covariance, t-SNE, t-distributed Stochastic Neighbor Embedding, ANN, Artificial Neural Network, ROC, Receiver Operator Characteristic, TPR, True positive rate, FPR, False positive rate

## Abstract

**Background:**

Breast cancer disease is the most common cancer in US women and the second cause of cancer death among women.

**Objectives:**

To compare and evaluate the performance and accuracy of the key supervised and semi-supervised machine learning algorithms for breast cancer prediction.

**Materials and methods:**

We have used nine machine learning classification algorithms for supervised (SL) and semi-supervised learning (SSL): 1) Logistic regression; 2) Gaussian Naive Bayes; 3) Linear Support vector machine; 4) RBF Support vector machine; 5) Decision Tree; 6) Random Forest; 7) Xgboost; 8) Gradient Boosting; 9) KNN. The Wisconsin Diagnosis Cancer dataset was used to train and test these models. To ensure the robustness of the model, we have applied K-fold cross-validation and optimized hyperparameters. We have evaluated and compared the models using accuracy, precision, recall, F1-score, and ROC curves.

**Results:**

The results of all models are inspiring using both SL and SSL. The SSL has high accuracy (90%–98%) with just half of the training data. The KNN model for the SL and logistic regression for the SSL achieved the highest accuracy of 98%

**Conclusion:**

The accuracies of SSL algorithms are very close to the SL algorithms. The accuracies of all models are in the range of 91–98%. SSL is a promising and competitive approach to solve the problem. Using a small sample of labeled and low computational power, the SSL is fully capable of replacing SL algorithms in diagnosing tumor type.

## Highlights

• Semi-supervised algorithms in integration with hyperparameters optimization yielded accurate prediction of breast cancer.
• Semi-supervised models take full advantage of the available information in the data and obtain the most accurate prediction.
• Semi-supervised algorithms can give very high accuracy (90%–98%) with just half of the training data.
• The KNN model for supervised and Logistic Regression for the semi-supervised learning have the most accurate prediction.

## 1 Introduction

Breast cancer usually arises in the ductal region and to a lesser extent in the lobules of the breast [1]. Breast cancer is the most common cancer in US women and is the second cause of cancer death among women. According to 2019 statistics of breast cancer, around268,600 new invasive cases were expected among US women, and 41,760 women were expected to die from this illness [2]. This disease incidence and mortality rates vary by race and age [1], however, it is highly curable when it is diagnosed early and before it metastasizes [3]. The diagnosis of breast cancer is very challenging and has a big attention worldwide due to the associated consequences of this disease as it has high morbidity and mortality rates [4]. The prediction of cancer category during its early stage has become an essential area in cancer research, as it can simplify the subsequent clinical requirements of patients and determines the effective treatments [5]. Early diagnosis of breast cancer can be a determining point between life and death [6]. The traditional technique to diagnose this cancer type is through using magnetic resonance imaging (MRI) and the microscopic examination of the tumor behavior to determine the tumor type and whether the tumor is malignant or benign. A benign tumor is a non-invasive type of tumor and it rarely causes life-threatening issues. On the contrast, a malignant tumors is an invasive kind that can affect the surrounding tissues and metastasize to distant tissues in the body. Modern approaches to the diagnosis of breast cancer use supervised learning (SL) to detect tumors with high accuracy [7].

With the advancement in the capabilities and state-of-the-art technologies of computer biomedical areas, numerous clinical tests and patient information related to breast cancer have been recorded. To control the rapid increase of breast cancer cases and minimize the risk factors, researchers have used the historical clinical records of patients to predict breast cancer [[8], [9], [10], [11], [12], [13]]. A variety of models have been developed to detect cancer using machine learning algorithms such as logistic regression, Decision Tree, Random Forest, eXtreme Gradient Boosting (Xgboost), etc [14]. In this study, we present broadly both SL and Semi-Supervised learning (SSL) aproaches. In SL, we have used labeled data to train the algorithm. Using large training data improves the supervised models’ performance. The SSL is a novel approach that uses a slight amount of labeled data to achieve very competitive results compared to the SL methods. The advantage of SSL is the fewer labeled data requirement and thereby avoiding the high cost of labeling. This study aims to compare and evaluate the performance and accuracy of the key SL and SSL algorithms for breast cancer prediction.

## 2 Material and methods

The main purpose of the machine learning techniques is to develop a classification model based on a given dataset that contains labeled classes and some attributes which include the dependent binary variable and independent variable. The process of the SL and SSL machine algorithms include mainly two steps: training and validation of the dataset. The algorithm uses the training dataset to adjust the predication model to minimize the error in the output results. The validation dataset is a split from the training dataset, which enables us to measure the progress of the learning algorithm independently. The main purpose of this measure is to determine end-point in the training algorithm to stabilize the accuracy trained model versus overfitting.

### 2.1 Supervised learning

SL is the most widely used machine learning technique. Machine learning requires learning of a function that fits the input pairs of values to output. The function extracts knowledge from labeled training data and each input pair corresponds to a labeled value. SL algorithms detect the pattern in the training data and produce a function that can predict new input pairs or never seen observations. The algorithm can generalize the function to predict the hidden accurately [15].

#### 2.1.1 Solving a problem using supervised technique

**Fig. 1:**
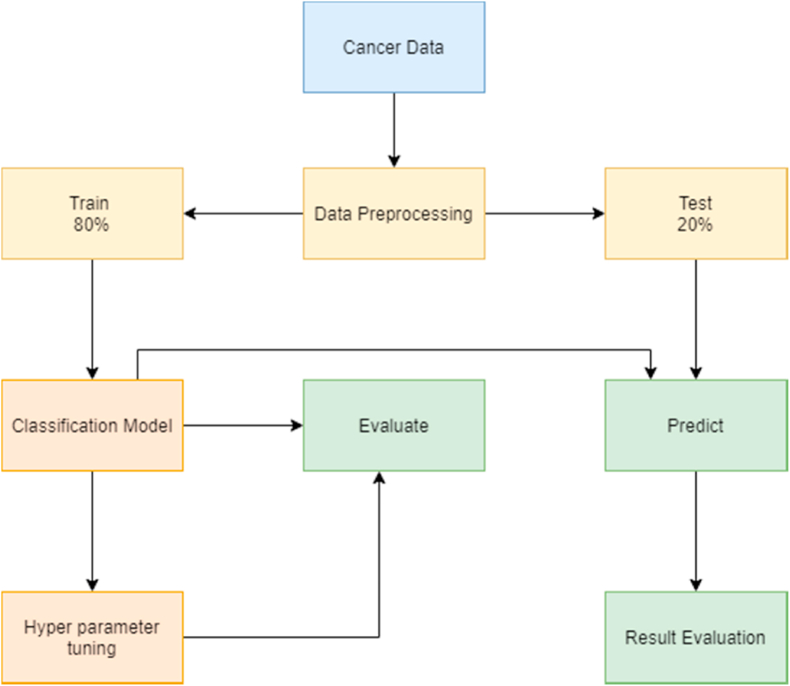
Supervised learning flowchart.

The SL algorithm solves problems by following/applying certain steps (Fig. 1):

1 Acquiring a dataset: The first step to solve any machine problem is to gather and collect the relevant data source. The data should be enough and have a sufficient number of rows and columns, as the size of the dataset depends on the problem we are solving [16].
2 Data processing: The dataset is cleaned by dealing with missing values, removing outliers, and normalizing the data. Data processing is the most crucial step in the machine learning process, as problems in the dataset will affect the accuracy of the prediction for the machine learning algorithm [17].
3 Identifying the type of target variable: The type of the targets variables determines a set of SL algorithms that can be applied. If the type of the variable is continuous, then it is a regression problem, and if the data type is categorical, then it is a classification problem. In this study, diagnosing cancer type as malignant or benign is a classification problem.
4 Splitting the dataset: The dataset is randomly split into training and test subsets. In this study, we have done an 80:20 split, with 80% of data for training and 20% for testing. We have ensured that both training and test contain balanced diagnosis values, so there is no problem of overfitting or underfitting.
5 Train the model: The training subset of the dataset is applied to the classification machine learning algorithm. We have applied nine classification machine learning algorithms and each algorithm is trained differently.
6 Hyperparameter tuning: Each algorithm can be optimized using a set of parameters. Training of algorithms begins with randomly initialized parameters and accuracies are evaluated accordingly. The parameters are optimized until a highest accuracy is achieved, then these parameters are used as a final machine learning algorithm to predict test data.
7 Prediction: The model is applied to the input data to predict the labels and results are evaluated accordingly based on the model outputs which include accuracy, precision, recall, f1-score, and support.

### 2.2 Semi-supervised learning

SL algorithms require a sizable amount of data to train the models with high prediction performance. In practical applications like medical diagnosis, image recognition, speech recognition, document classification, there is an enormous amount of unlabeled data available which hinders the model to incorporate unlabeled data. This obstacle can be overcome by using an SSL algorithm.

SSL is considered as a hybrid approach of SL and unsupervised learning. The algorithm is provided with unlabeled data along with the supervision information in a small quantity. The output of SSL contains target variables that are used to train and predict the targets for the unlabeled data.

Algorithm 1 Semi-supervised learning algorithm

Input: Labeled data$\;{{\left\{\left(X_{i},y_{i}\right)\right\}}^{l}}_{i=1}$ , unlabeled data ${{\left\{X_{j}\right\}}^{l+u}}_{j=l+1}$;

1. *Initialize:* let *L* = ${{\left\{\left(X_{i},y_{i}\right)\right\}}^{l}}_{\;i=1}$ and *U* = ${{\left\{X_{j}\right\}}^{l+u}}_{j=l+1}$
2. Normalize *L* = ${{\left\{\left(X_{i},y_{i}\right)\right\}}^{l}}_{\;i=1}$ and *U* = ${{\left\{X_{j}\right\}}^{l+u}}_{j=l+1}$
3. Repeat:
4. Train *f* from L using supervised machine learning algorithm.
5. Apply f to the unlabeled instances in U.
6. Remove a subset *S* from U; add {(X,f(x))|X∈S} to *L.*

#### 2.2.1 Solving the problem using semi-supervised learning algorithm

**Fig. 2:**
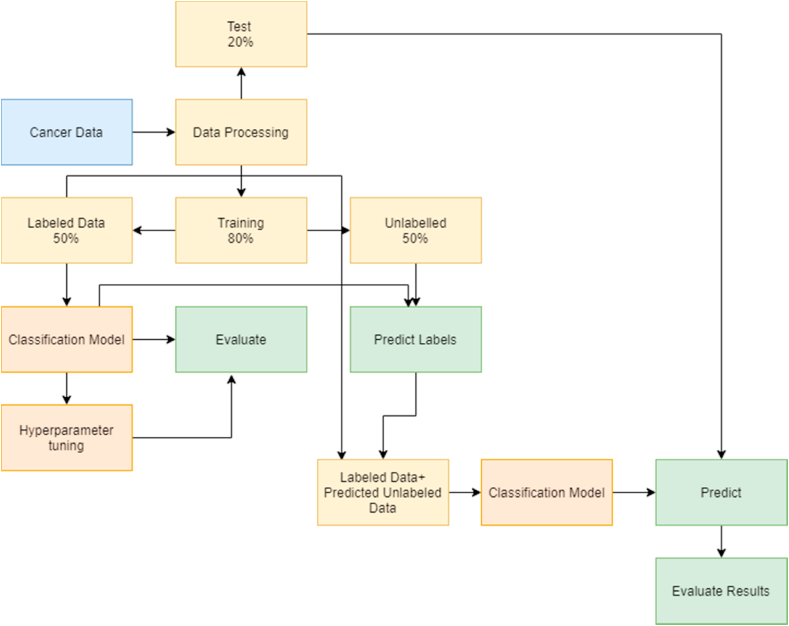
Semi-supervised learning flowchart.

SSL algorithm solves problems by following/applying certain steps (Fig. 2):

1. Data processing- Input featured are normalized to make all variables on the same scale and distribution. In this study, we have used only 50% of the train data to fit the machine learning algorithm and 50% of train data as unlabeled data.
2. Labeled and Unlabeled data- The main advantage of the SSL algorithm is to have unlabeled data and a smaller amount of labeled data. Herein, we divided 80% of the training data into 50% labeled data by including the target variable and 50% unlabeled data by removing the target variable. In real scenarios, there is a huge amount of unlabeled data as labeling data is expensive and time-consuming. Therefore, there is no need to remove the target variables to create unlabeled data. The SSL approach can be applied using the small training dataset and the large unlabeled data to train the algorithm.
3. Train the model- The model is trained by 80% of the data and half of it was unlabeled. We have nine classification algorithms that are trained and optimized using hyperparameter optimization.
4. Hyperparameter optimization – The model gets the highest accuracy by randomly initializing the parameters and changing them until the highest accuracy is achieved.
5. Predicting Labels for the unlabeled data- Labels for the unlabeled data are predicted and combined with labeled data. This creates a large dataset and then the model is again trained with hyperparameter optimization.
6. Predicting the labels for the test data – Finally, the test data is predicted by using the trained model and results are evaluated according to the accuracy, precision, recall, f1-score, and support.

By using this SSL classification we reduce the usage of the training data [18].

### 2.3 Statistical analysis

Statistical packages Python version 3.7.5 was used to analyze the dataset. A descriptive analysis was used in describing the basic features of the dataset in the study area (malignant and benign). The study was registered with the Research Registry (researchregistry6268) in accordance with the declaration of Helsinki. The study was conducted according to the guidelines of Strengthening the reporting of cohort studies in surgery (STROCSS) 2019 [20].

### 2.4 Data processing and evaluation

#### 2.4.1 Dataset

We have used Wisconsin Diagnostic Breast Cancer (WDBC) dataset [19] to apply the machine learning algorithms. The dataset consists of patient ID, cell nuclei features, and diagnosis. The ID is the patient identification number, and the cell nuclei features were determined from a digital image of a fine needle aspirate (FNA) of a breast mass. These features describe 10 characteristics of each cell nucleus (Table 1).

**Table 1:** Features of breast cancer data.

Breast Cancer Data Characteristics	Description
Radius	Mean of distances from center to points on the perimeter
Texture	standard deviation of gray-scale values
Perimeter	Perimeter of tumor
Area	Area of tumor
Smoothness	local variation in radius lengths
Compactness	Perimeter^2/area - 1.0
Concavity	Severity of concave portions of the contour
Concave points	Number of concave portions of the contour
Fractal	“Coastline approximation” – 1

Each of these characteristics consists of three features: (1) mean, (2) standard error (3) worst. So, a total of 30 features of 569 patients were evaluated. Of all cases, there are 357 benign cases and 212 malignant ones.

#### 2.4.2 Data exploration

**Fig. 3:**
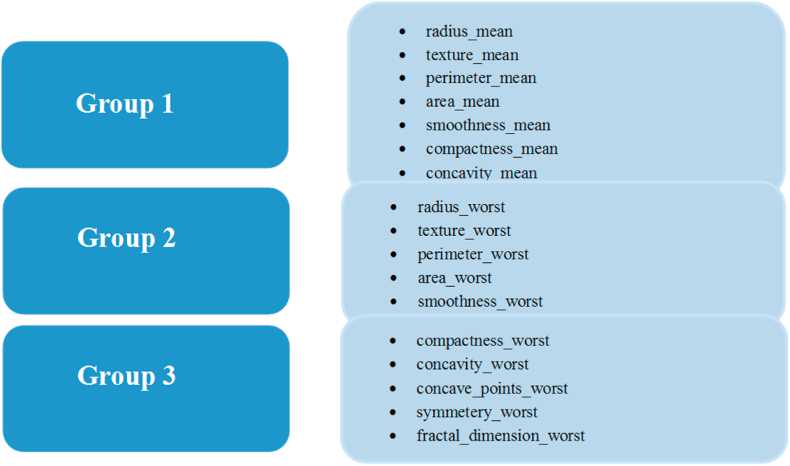
The three major groups of studied features.

There are many features and analyzing all the features will not give a clear picture and insights. Therefore, the features were divided into three major groups to explore relations among them (Fig. 3).

To analyze all the groups, we implemented pair plots for each of these groups.

#### 2.4.3 Pair plots

Pair plots come under Exploratory Data Analysis (EDA). EDA is the process of finding the patterns and relationships existing in the data. Pair plots are one of the useful EDA tools to visualize the relationships. Pair plots are also called the Scatter matrix plot. Pair plots enable us to evaluate the distribution of a single variable, determine the relationships between two variables, and to find trends that can be used in further analysis.

##### 2.4.3.1 Pair plots of group 1

**Fig. 4:**
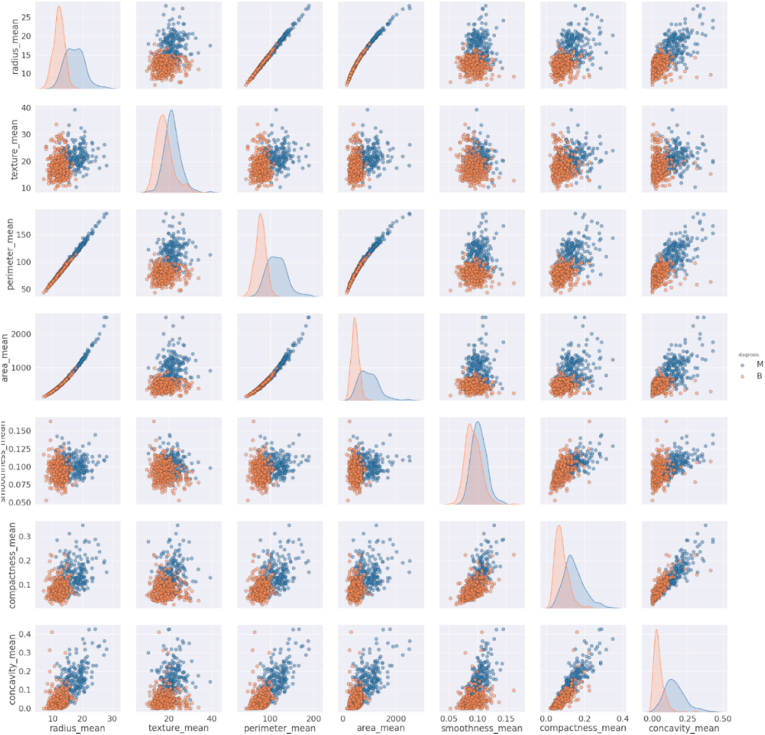
Pair plot of Group 1 features.

As shown in Fig. 4, the texture_mean and smoothness_mean, are normally distributed, but other features are not. We can see a positive correlation in the scatter plots of radius_mean with area_mean & perimeter_mean and between compactness_mean & concavity_mean. The figures also explain the malignant breast cancer for high values of the features. The lower portion of all scatter plot is occupied for benign while the upper portion is for malignant. The increase in the size of these features indicates that the tumor is malignant.

##### 2.4.3.2 Pair plots of group 2

**Fig. 5:**
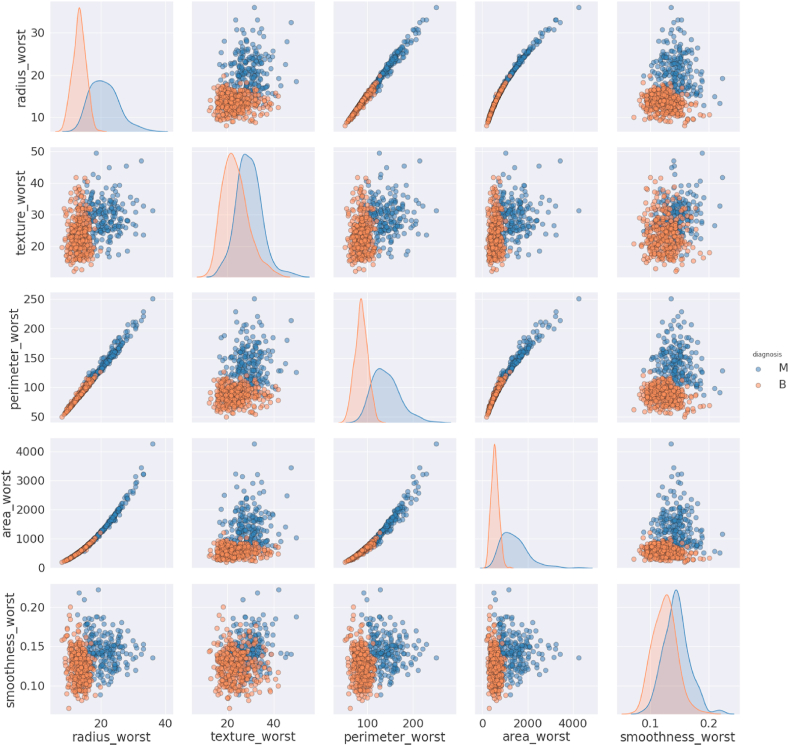
Pair plot of Group 2 features.

The features texture_worst and smoothness_worst are normally distributed, but others are not for both of the diagnosis codes. There is an upward linear relationship between radius_worst and perimeter_worst, radius_worst and area_worst, and perimeter_worst and area_worst (Fig. 5). Other relationships have no clear indicator that the increase in the size of features will indicate the diagnosis as malignant.

##### 2.4.3.3 Pair plots of group 3

**Fig. 6:**
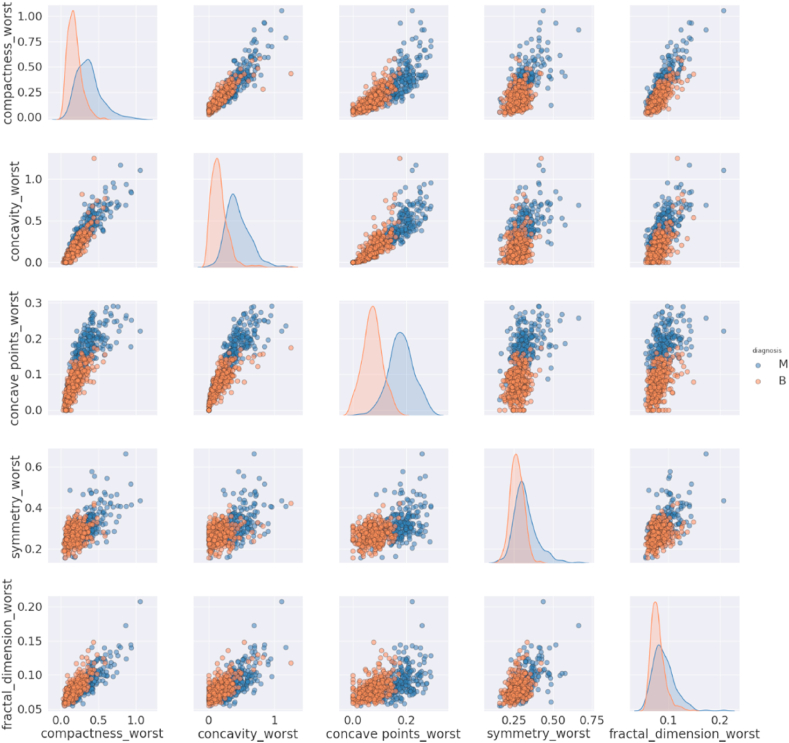
Pair plot of Group 3 features.

There is no linear relationship between the variables of group 3 and the diagnoses are mixed (Fig. 6). We can see from the above pair plots that some of the features are correlated, but some are not because they represent different characteristics and do not have a relation with others. We applied correlation analysis to check the significant relationship between the variables.

**Fig. 7:**
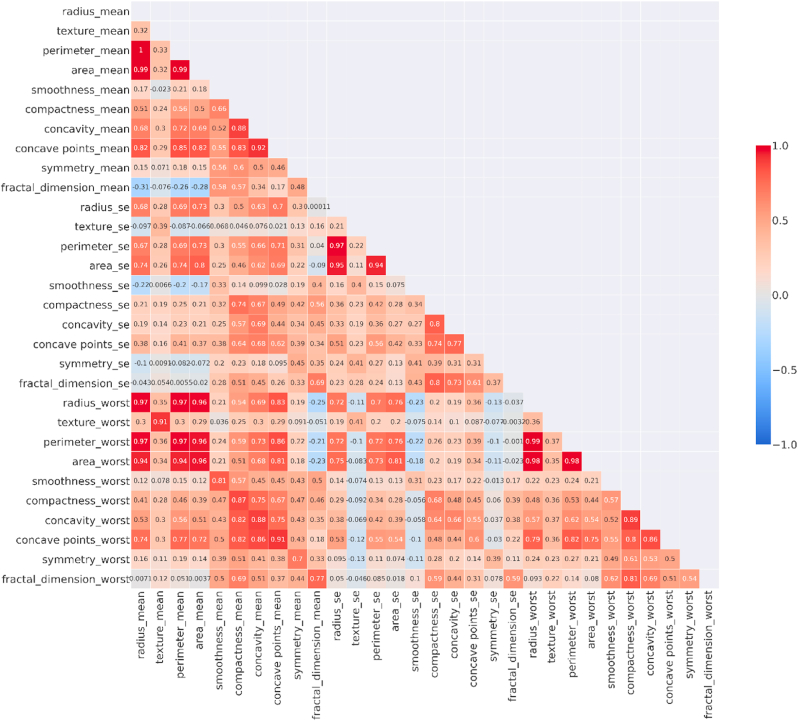
Heat map representing correlation values for the input features.

We have calculated the Pearson coefficient for each pair of features and converted it into a heat map (Fig. 7).

The insights we have obtained from the pair plots are confirmed by the heat map. There is a strong positive correlation between perimeter_worst and radius_mean, area_worst, and radius_mean.

Some of the features are highly correlated. This could mean that these features can represent the same thing and should be removed before applying any classification algorithm. This can only be justified by visualizing all the features in two-dimensions.

In this study, t-SNE visualization was implemented to visualize the feature space by diagnosis code in two-dimensions.

### 2.5 t-SNE (t-distributed stochastic neighbor embedding)

t-SNE is an unsupervised machine learning algorithm that finds the pattern in the data, and a non-linear dimensionality reduction technique unlike PCA for reducing and visualizing high dimensional space into two or three dimensions. t-SNE selects two similarity measures between pairs of points - one measure for the high dimensional data and another for the two-dimensional embedding. Next, it tries to build a two-dimensional embedding that reduces the Kullback–Leibler divergence between the vector of similarities between pairs of points in the original dataset and the likeness between pairs of points in the embedding. At a high level, t-SNE starts with an embedding that is randomly started and makes repeated gradual updates to it. Thus, the analysis evaluates the effect of this update to the embedding of the high-dimensional points in terms of if they lie in the same cluster or not [21]. The t-SNE algorithm consists of two main stages:

1. t-SNE builds a probability distribution over pairs of high-dimensional objects in a manner that alike points have a high probability to be selected while dissimilar points have a particularly small probability to be selected.
2. t-SNE describes a probability distribution over the points in the low-dimensional space, and it reduces the Kullback–Leibler divergence between the two probability distributions for the locations of the points in the space.

**Fig. 8:**
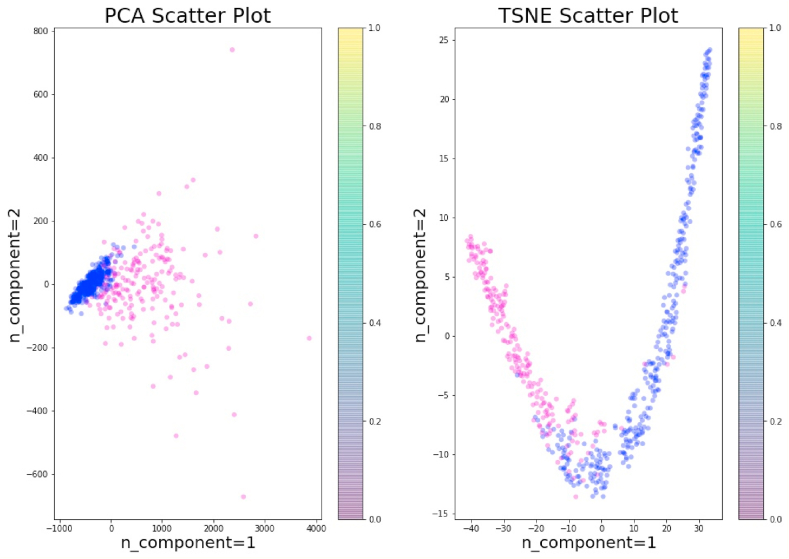
PCA and t-SNE without standardization.

**Fig. 9:**
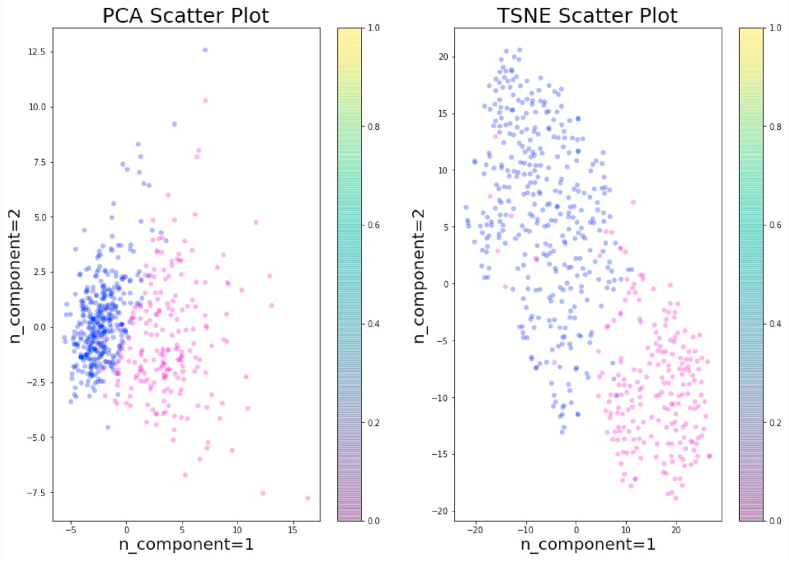
PCA and t-SNE with Standardization.

The t-SNE has been applied for visualization in many applications, including cancer diagnosis, biomedical field, bioinformatics, etc. It is mostly used for the visualization of high-level representations learned by an artificial neural network (ANN). In this study, we have applied PCA and t-SNE with two components to visualize the data before (Fig. 8) and after standardization (Fig. 9).

## 3 Results

### 3.1 Evaluation

We have applied both SL and SSL techniques for nine classification machine learning algorithms. Evaluation is done by randomly sampling 20% of the breast cancer data as a test sample (Table 2).

**Table 2:** Summary of classification algorithms.

Methods	Measures	Precision		Recall		F1-Score	
		SL	SSL	SL	SSL	SL	SSL
Decision Tree	Malignant %	88%	97%	88%	83%	88%	90%
	Benign %	93%	91%	93%	99%	93%	95%
	Accuracy %	91%	93%	91%	93%	91%	93%
	Avg %	91%	94%	91%	91%	91%	92%
Gradient Boosting	Malignant %	91%	91%	93%	93%	92%	92%
	Benign %	96%	96%	94%	94%	95%	95%
	Accuracy %	94%	94%	94%	94%	94%	94%
	Avg %	93%	93%	94%	94%	93%	93%
Gaussian Naïve Bayes	Malignant %	93%	88%	93%	86%	93%	87%
	Benign %	96%	92%	96%	93%	96%	92%
	Accuracy %	95%	90%	95%	90%	95%	90%
	Avg %	94%	90%	94%	89%	94%	90%
KNN	Malignant %	98%	95%	98%	98%	98%	96%
	Benign %	99%	99%	99%	97%	99%	98%
	Accuracy %	98%	97%	98%	97%	98%	97%
	Avg %	98%	97%	98%	97%	98%	97%
Logistic Regression	Malignant %	100%	100%	93%	95%	96%	98%
	Benign %	96%	97%	100%	100%	98%	99%
	Accuracy %	97%	98%	97%	98%	97%	98%
	Avg %	98%	99%	96%	98%	97%	98%
Random Forest	Malignant %	93%	95%	95%	95%	94%	95%
	Benign %	97%	97%	96%	97%	96%	97%
	Accuracy %	96%	96%	96%	96%	96%	96%
	Avg %	95%	96%	96%	96%	95%	96%
SVM Linear	Malignant %	95%	100%	98%	93%	96%	96%
	Benign %	99%	96%	97%	100%	98%	98%
	Accuracy %	97%	97%	97%	97%	97%	97%
	Avg %	97%	98%	97%	96%	97%	97%
SVM RBF	Malignant %	98%	100%	93%	93%	95%	96%
	Benign %	96%	96%	99%	100%	97%	98%
	Accuracy %	96%	97%	96%	97%	96%	97%
	Avg %	97%	98%	96%	96%	96%	97%
Xgboost	Malignant %	98%	95%	95%	86%	96%	90%
	Benign %	97%	92%	99%	97%	98%	95%
	Accuracy %	97%	93%	97%	93%	97%	93%
	Avg %	97%	93%	97%	91%	97%	92%

SL: Supervised Learning, SSL: Semi Supervised Learning.

All the algorithms performed well on the test data. There is no substantial difference in accuracies of SL and SSL technique. The Logistic Regression (SL = 97% and SSL = 98%) and KNN (SL = 98% and SSL = 97%) are best performing algorithms in all the measures. These two algorithms have a very high prediction of both malignant and benign tumors. Logistic regression is 100% correct in predicting the malignant category for SL and SSL. KNN is 98–99% correct in predicting both malignant and benign. The precision, Recall, and F1-scores of Logistic Regression and KNN shows that the algorithm is neither over nor under fitted. Therefore, it can be concluded that the accuracies of these algorithms are reliable. Further, all machine learning algorithms that have been used in this study do not suffer from under-fitting or overfitting. The average is also highest for Logistic Regression (SL = 98% and SSL = 99%) and KNN (SL = 98% and SSL = 97%). Interestingly, the SSL approach was better than SL for decision trees (SL = 91% and SSL = 94%). The results of SSL are very close to the SL. The only exception is Xgboost (SL = 97% and SSL = 93%) as it requires many rows to achieve good accuracy. For the problem of breast cancer diagnosis, the SSL techniques can replace SL techniques because of the high accuracy and reliability with fewer data and computation.

In summary, the rate of detection of breast cancer is excellent for KNN, Logistic Regression, and SVM Linear. Further, the Logistic Regression, SVM Linear, and SVM RBF reached 100% accuracy in breast diagnosis categories in SSL. Only Gaussian Naïve Bayes is less than 90% in detecting breast cancer. The SSL models are performing better than SL models in terms of sensitivity and specificity Table 3.

**Table 3:** Sensitivity and Specificity of algorithms.

Methods	Sensitivity		Specificity	
	SL	SSL	SL	SSL
Decision Tree	88.00%	97.00%	93.00%	99.00%
Gradient Boosting	93.00%	91.00%	94.00%	94.00%
Gaussian Naïve Bayes	93.00%	88.00%	96.00%	93.00%
KNN	98.00%	95.00%	99.00%	97.00%
Logistic Regression	93.00%	100.00%	100.00%	100.00%
Random Forest	95.00%	95.00%	96.00%	97.00%
SVM Linear	98.00%	100.00%	97.00%	100.00%
SVM RBF	93.00%	100.00%	99.00%	100.00%
Xgboost	95.00%	95.00%	99.00%	97.00%

#### 3.1.1 ROC curve and confusion matrix

A Receiver Operator Characteristic (ROC) curve is a visual representation used to explain the diagnostic capability of binary classifiers. The ROC curve reveals the sensitivity -true positive rate (TPR) and specificity (1 – false positive rate (FPR)). Classifiers that provide curves closer to the top-left corner represent a reliable performance. As a baseline, a random classifier is required to put up points along the diagonal line (FPR = TPR). The nearer the curve reaches the 45-degree diagonal of the ROC area, the less accurate the test.

We have plotted the ROC curves and the Confusion matrices for all the algorithms. ROC curves and Confusion matrices for all the algorithms are almost perfect, and algorithms are accurate in distinguishing between malignant and benign lesions (Table 4).

**Table 4:** Area under the curve (AUC) of ROC curves.

Model	AUC of ROC curve Supervised	AUC of ROC curve Semi-supervised
Decision tree	0.89	0.9
Gaussian Naive Bayes	0.94	0.89
Logistic Regression	0.96	0.98
Random Forest	0.96	0.96
Xgboost	0.97	0.91
KNN	0.98	0.97
SVM	0.97	0.96
RBF SVM	0.96	0.96
Gradient Boosting Machine	0.98	0.92

#### 3.1.2 Precision and recall curve

**Fig. 10:**
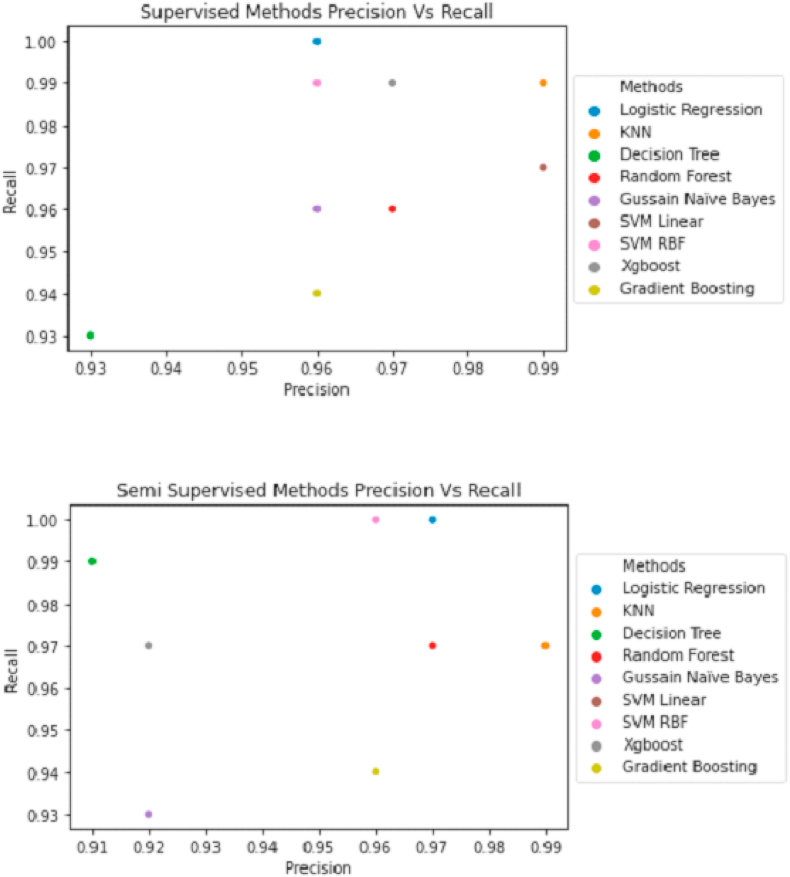
Precision vs. Recall curve for supervised and semi supervised learning.

The precision versus recall curve shows that Logistic Regression and KNN are reliable for predicting breast cancer (Fig. 10), as observed in our previous findings of the evaluation of algorithms.

## 4 Discussion

A variety of models have been developed to detect cancer using machine learning algorithms such as logistic regression, Decision Tree, Random Forest, Xgboost, etc [14]. Machine learning has the capabilities of probabilistic, statistical, and optimization techniques which play a vital role in cancer diagnosis. Hence, the precise prediction of machine learning techniques for cancer diagnosis has become one of the most crucial and inspiring errands for researchers [22]. Three types of machine learning methods are commonly used: SL, unsupervised learning, and SSL. In SL, the labeled training data is linked to the targeted output. In unsupervised machine learning, unlabeled training data is used to find groups of alike samples or patterns. While in SSL, both labeled and unlabeled data is employed to create an accurate model [4]. Nevertheless, in most cancer prediction, researchers consider only labeled data while ignoring most of unlabeled data. In this study, SL and SSL classification algorithms were applied by utilizing labeled and unlabeled information. The results show that the proposed SSL models use the available information in the data and obtain the most accurate prediction.

Ubaidillah et al. used neural network (NN) models and the SVM on the dataset of the BUPA liver disorders and results revealed that the SVM classifier has more reliable performance than the NN for classifying liver cancer [23]. Statnikov et al. applied and compared the random forest and SVM methods on 22 diagnostic and prognostic datasets. The results demonstrated that by using the full set of genes, SVMs showed better performance than RFs often by a large margin “fifteen datasets”, while the RFs method showed better performance compared to SVMs on four datasets and both methods showed similar performance on three datasets. Similar results were obtained using the selected genes [24]. Alireza et al. applied SVM classification technique on two different clinical datasets for breast cancer and SL algorithm yielded 98.80% and 96.63% accuracies [25]. In line with these studies, our results showed that SVM RBF has a precision of 98% in detecting malignant tumors, while SVM linear has 95%, and RF has 93% in SL. However, these algorithms have a higher precision in the SSL as 100% of SVM and 95% for RF. Further, SVM RBF, SVM linear, and RF have accuracies as 96%, 97%, and 96%respectively in SL and as 97%, 97%, and 96% in SSL.

Haifeng et al. applied different SL algorithms Naive Bayes Classifier, SVM, AdaBoost tree, ANN, they have used a hybrid between principal component analysis (PCA) and related data mining models, which applies a PCA for dimensionality reduction to find an effective way for breast cancer prediction [26]. Karabatak et al. applied a hybrid model to detect breast cancer, the association rule, and Neural Network (NN) hybrid was used. In the model, the association rule was used along with the NN to reduce the dimension of feature space of the breast cancer database and for brilliant classification, respectively. The proposed prediction model was verified using the Wisconsin breast cancer database. The results showed that the hybrid model algorithm has increased the efficiency and the accuracy of automatic diagnostic systems [27]. On the other hand, many researchers have applied the Bayesian classifiers in studies that heavily rely on the probabilistic based classification technique [8,28,29]. We have used the Gaussian Naïve Bayes which showed an accuracy of 95% in SL and 90% for SSL. Besides, the fuzzy algorithm was discussed in predicting breast cancer [[30], [31], [32]]. A study presented a hybrid approach through combining the fuzzy systems and evolutionary algorithm [32]. In the research, a fuzzy genetic algorithm was applied to the Wisconsin breast cancer diagnosis database. The results showed that the proposed fuzzy genetic model can provide explicable results with high classification performance.

Aisl algorithm is a novel automated algorithm for cancer diagnosis. It integrates artificial immune with SSL learning (Aisl). Since it is a SSL, Aisl can deal with both the labeled and unlabeled data. In addition, it applies the adaptability of the immune system and it proved its effectiveness and efficiency on two famous UCI breast cancer datasets with an accuracy of 98.0% and a precision of 95.9% [33]. In our study, the SSL algorithms Logistic Regression obtained and KNN have accuracies of 98% and 97% and precisions of 99% and 97% respectively.

On the other hand, other researchers developed an algorithm that used pseudo labels for the data. They used a convolutional neural network-based model that is validated on PatchCamelyon (PCam) benchmark dataset for fundamental machine learning research in histopathology diagnosis of cancer metastasis. The results showed a better performance of this model to detect metastasis [34].

## 5 Conclusion

This study concluded that the accuracies of SSL algorithms are very close to SL algorithms. The two best-performing algorithms are KNN (SL = 98% & SSL = 97%) and logistics regression (SL = 97% & SSL = 98%). The accuracies of all models are in the range of 91–98%. We did not observe any overfitting and underfitting, as the predictions were accurate for both malignant and benign tumors. SSL proves to be a promising and competitive approach to solve the problem. Using a small sample of labeled and low computational power, SSL is fully capable of replacing SL algorithms in diagnosing tumor type. Though we have achieved the highest accuracy of 98% in this study, future work can be carried out to remove the chance of the 2% error of incorrect predicted diagnosis by using deep learning methods and applying different data processing and feature engineering.

## Funding

No funding received.

Provenance and peer review.

Not commissioned, externally peer reviewed.

## Annals of medicine and surgery

The following information is required for submission. Please note that failure to respond to these questions/statements will mean your submission will be returned. If you have nothing to declare in any of these categories then this should be stated.

Please state any conflicts of interest.

All authors must disclose any financial and personal relationships with other people or organisations that could inappropriately influence (bias) their work. Examples of potential conflicts of interest include employment, consultancies, stock ownership, honoraria, paid expert testimony, patent applications/registrations, and grants or other funding.

Authors declare no conflict or competing interest.

Please state any sources of funding for your research.

All sources of funding should be declared as an acknowledgement at the end of the text. Authors should declare the role of study sponsors, if any, in the collection, analysis and interpretation of data; in the writing of the manuscript; and in the decision to submit the manuscript for publication. If the study sponsors had no such involvement, the authors should so state.

No funding was received.

## Ethical approval

Research studies involving patients require ethical approval. Please state whether approval has been given, name the relevant ethics committee and the state the reference number for their judgement.

We have used The Wisconsin Breast Cancer dataset. The dataset was obtained from the University of Wisconsin Hospitals, Madison from Dr. William H. Wolberg. The Forest Covertype is Copyrighted 1998 by Jock A. Blackard and Colorado State University.

## Consent

Studies on patients or volunteers require ethics committee approval and fully informed written consent which should be documented in the paper.

Authors must obtain written and signed consent to publish a case report from the patient (or, where applicable, the patient's guardian or next of kin) prior to submission. We ask Authors to confirm as part of the submission process that such consent has been obtained, and the manuscript must include a statement to this effect in a consent section at the end of the manuscript, as follows: “Written informed consent was obtained from the patient for publication of this case report and accompanying images. A copy of the written consent is available for review by the Editor-in-Chief of this journal on request”.

Patients have a right to privacy. Patients’ and volunteers' names, initials, or hospital numbers should not be used. Images of patients or volunteers should not be used unless the information is essential for scientific purposes and explicit permission has been given as part of the consent. If such consent is made subject to any conditions, the Editor in Chief must be made aware of all such conditions.

Even where consent has been given, identifying details should be omitted if they are not essential. If identifying characteristics are altered to protect anonymity, such as in genetic pedigrees, authors should provide assurance that alterations do not distort scientific meaning and editors should so note.

Not applicable.

## Author contribution

Please specify the contribution of each author to the paper, e.g. study concept or design, data collection, data analysis or interpretation, writing the paper, others, who have contributed in other ways should be listed as contributors.

Nosayba Al-Azzam proposed the concept, designed the methodology, and wrote the manuscript.

Ibrahem Shatnawi carried out the calculations and analysis and wrote the manuscript.

Registration of Research Studies.

In accordance with the Declaration of Helsinki 2013, all research involving human participants has to be registered in a publicly accessible database. Please enter the name of the registry and the unique identifying number (UIN) of your study.

You can register any type of research at http://www.researchregistry.com to obtain your UIN if you have not already registered. This is mandatory for human studies only. Trials and certain observational research can also be registered elsewhere such as: ClinicalTrials.gov or ISRCTN or numerous other registries.

1. Name of the registry:

Research registry.

2. Unique Identifying number or registration ID:

Researchregistry6268.

3. Hyperlink to your specific registration (must be publicly accessible and will be checked):

https://www.researchregistry.com/browse-the-registry#home/

## Guarantor

The Guarantor is the one or more people who accept full responsibility for the work and/or the conduct of the study, had access to the data, and controlled the decision to publish.

## Declaration of competing interest

Authors declare no conflict or competing interest.
